# Innovations in research ethics governance in humanitarian settings

**DOI:** 10.1186/s12910-015-0002-3

**Published:** 2015-02-26

**Authors:** Doris Schopper, Angus Dawson, Ross Upshur, Aasim Ahmad, Amar Jesani, Raffaella Ravinetto, Michael J Segelid, Sunita Sheel, Jerome Singh

**Affiliations:** Medical Faculty, University of Geneva, Geneva, Switzerland; Centre for Education and Research in Humanitarian Action (CERAH), Geneva, Switzerland; Centre for Biomedical Ethics, School of Health and Population Sciences, University of Birmingham, Birmingham, UK; Department of Family and Community Medicine, University of Toronto, Toronto, Canada; The Kidney Centre, Karachi, Pakistan; Aga Khan University, Karachi, Pakistan; Anusandhan Trust, Mumbai, India; Department of Clinical Sciences, Institute of Tropical Medicine, Antwerp, Belgium; Department of Pharmaceutical and Pharmacological Sciences, Leuven, KU Belgium; Centre for Human Bioethics, Monash University, Clayton, Australia; Independent researcher in global health and bioethics, Pune, India; Centre for the AIDS Programme of Research in South Africa (CAPRISA), Durban, South Africa

**Keywords:** Research ethics, Humanitarian contexts, Framework for ethics review, Research impact

## Abstract

**Background:**

Médecins Sans Frontières (MSF) is one of the world’s leading humanitarian medical organizations. The increased emphasis in MSF on research led to the creation of an ethics review board (ERB) in 2001. The ERB has encouraged innovation in the review of proposals and the interaction between the ERB and the organization. This has led to some of the advances in ethics governance described in this paper.

**Discussion:**

We first update our previous work from 2009 describing ERB performance and then highlight five innovative practices:

• A new framework to guide ethics review

• The introduction of a policy exempting a posteriori analysis of routinely collected data

• The preapproval of “emergency” protocols

• General ethical approval of “routine surveys”

• Evaluating the impact of approved studies

The new framework encourages a conversation about ethical issues, rather than imposing quasi-legalistic rules, is more engaged with the specific MSF research context and gives greater prominence to certain values and principles. Some of the innovations implemented by the ERB, such as review exemption or approval of generic protocols, may run counter to many standard operating procedures. We argue that much standard practice in research ethics review ought to be open to challenge and revision. Continued interaction between MSF researchers and independent ERB members has allowed for progressive innovations based on a trustful and respectful partnership between the ERB and the researchers. In the future, three areas merit particular attention. First, the impact of the new framework should be assessed. Second, the impact of research needs to be defined more precisely as a first step towards being meaningfully assessed, including changes of impact over time. Finally, the dialogue between the MSF ERB and the ethics committees in the study countries should be enhanced.

**Summary:**

We hope that the innovations in research ethics governance described may be relevant for other organisations carrying out research in fragile contexts and for ethics committees reviewing such research.

## Background

Médecins Sans Frontières (MSF) is one of the world’s leading humanitarian medical organizations. It provides emergency medical assistance to populations in danger in more than 70 countries. The foundational and animating values of MSF as a humanitarian medical organization are rooted in ethics [1]. Historically, research was not seen as core to the mission of MSF. However, during its history, MSF has constantly developed innovative protocols and tools, in response to unmet field needs [2]. It now initiates, sponsors or participates in numerous research projects in multiple field sites. In 2013 alone, 253 research papers were published [3]. The results of MSF research have had substantial impact on global health policy and provide benefits to populations served by MSF and elsewhere as presented later in this paper. MSF has also shown leadership in operational research initiatives in the humanitarian NGO sector [4]. As a result, research has become increasingly integral to MSF activities, both in the field and in global health advocacy. The increased emphasis on research led to the creation of an ethics review board (ERB) in 2001. From the outset independence of the ERB from the organisation was perceived as essential. To avoid conflict of interest and ensure independence, ERB members cannot have a working relationship with MSF during their tenure. The ERB has now been in place for thirteen years. The ERB’s dialogue with MSF has enhanced sensitivities towards research ethics and lead to gradual improvements in developing ethically sound proposals. The consistent use of an ethics framework has helped in standardizing the review process and guiding field research teams in addressing ethical issues. The way that the ERB functions and the challenging ethical issues addressed by the ERB since its inception have been described previously [5]. The ERB strives to take a constructive approach to both research proposals and the field of research ethics itself. The ERB has encouraged innovation in both the review of proposals and the interaction between the ERB and the organization.

**Figure 1:**
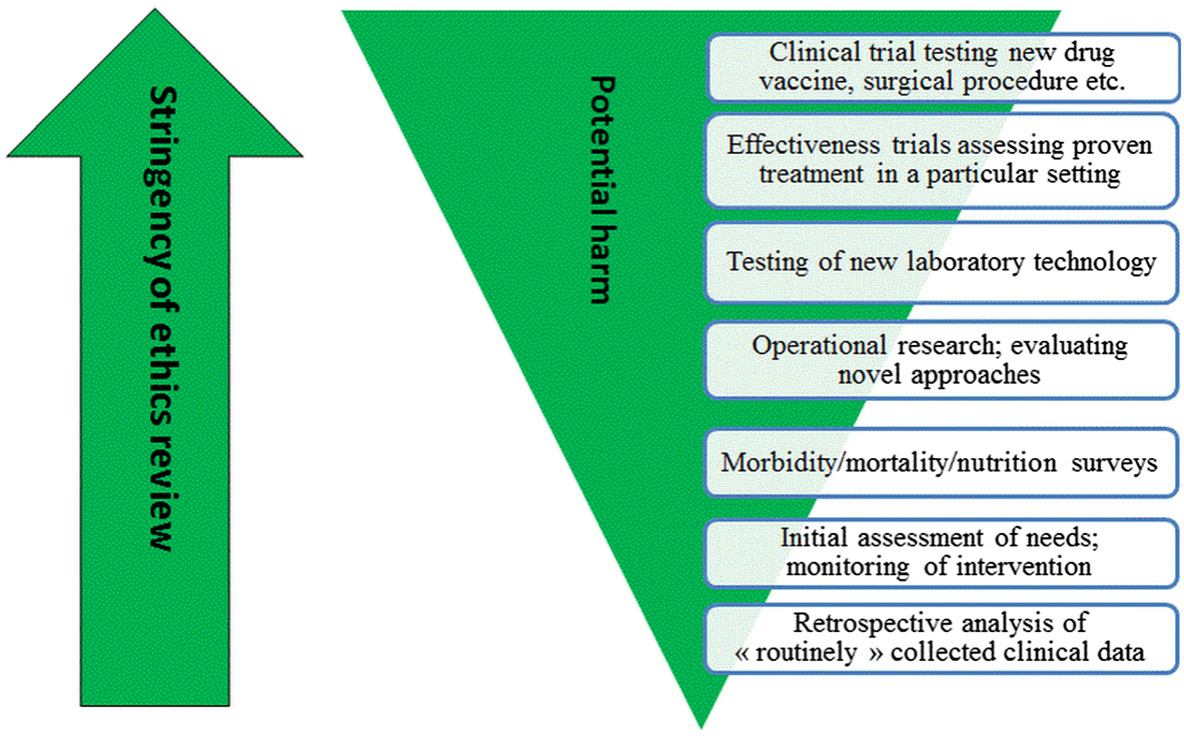
**Proportionality of ethics review.**

In the view of the ERB ethics review should not be dogmatic and ought to be proportionate in terms of benefit and harms. The ethics review procedure and its stringency should be commensurate with the type of research based on an approximate estimation of potential harm (Figure 1).

This has led to some of the advances in ethics governance further described in this paper. Some of the innovations implemented by the ERB may run counter to standard operating procedures and guidance documents that are utilized in various jurisdictions. We argue that much standard practice in research ethics review ought to be open to challenge and revision, and this irrespectively of the actors and the context of the research. Research ethics is a field that should not be regarded as a set of rigid standards fixed for all time and in all contexts. Much of current research ethics practice lacks an empirical basis, and many recommended standards are based on historical practice. From our perspective, research ethics and research ethics practices are capable of being examined through research and a quality improvement approach. Any genuine ethics review requires critical reflection and discussion, not the pedestrian adoption of legalistic rules, and it should result in a learning process for both the researchers and the reviewers. Fostering a spirit of innovation and evaluation in research ethics practice demonstrates that research ethics itself is an active and essential component of global health research. In this paper we describe innovations that we think challenge accepted practices and thus hope to stimulate a more vigorous debate in this area, beyond the realm of research carried out by MSF.

To contextualise our proposals for changes in research ethics governance, we will first update our previous work from 2009 [5] describing ERB performance and then highlight five innovative practices:

- A new framework to guide ethics review
- The introduction of a policy exempting *a posteriori* analysis of routinely collected data
- The preapproval of “emergency” protocols
- General ethical approval of “routine surveys”
- Evaluating the impact of approved studies

## Discussion

### ERB performance

**Figure 2:**
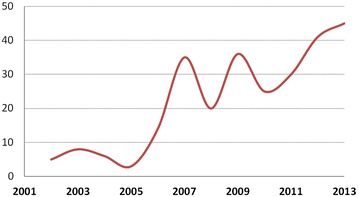
**Reviews since ERB inception.**

Before describing the five areas of innovation, we will briefly give an overview of ERB activities and performance focusing on the past four years. Since its inception, the volume of ERB activity has increased considerably, as reflected in the number of protocols received. Overall 248 research protocols were reviewed by the ERB between 2002 and 2013 with an almost ten-fold increase in the number of protocols across that period (Figure 2). Protocols are always submitted to the ERB and to the Ethics Committees in the study country, and the approval from the local Ethics Committee is a fundamental prerequisite to the ERB approval. In very rare instances, the ERB may waive this request if, for example, no ethics committee or national body able to play a similar function is available, or if it is justified not to collaborate with the legal authorities, as this could be detrimental to the population served by MSF (e.g. working in a conflict zone with a population group persecuted by national authorities).

In 2010–2011, 56 requests for review were received in 24 months. This increased to 72 requests during the two-year period 2012–13, despite the fact that during this time research based on a posteriori analyses of routinely collected clinical data was no longer submitted to the ERB for review. Of this number 61 were expedited reviews and 11 were full board reviews^a^.

**Figure 3:**
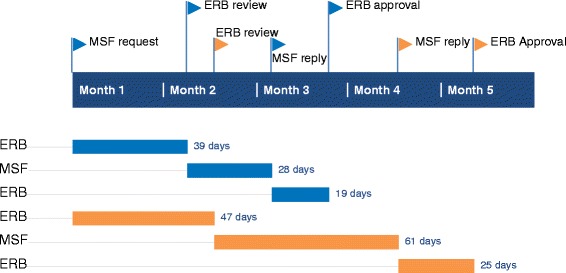
**Average time procedure for expedited review (in blue) and full review (in orange).**

The Board is also evaluating the timeliness of its reviews. An audit of proposals from 2010–2013 (four-year period) demonstrated an average time to approval of twelve weeks (Figure 3).

Figure 3 shows that, on average, the ERB needs more time for doing the first review (time between MSF request and finalizing ERB review) than researchers for responding (time between ERB review and MSF reply) in the case of expedited review. However, the ERB is faster than the investigators in case of full review. Once a satisfactory reply and revisions from the researchers are received, approval is usually granted quickly. Overall the procedure is faster for expedited review than for full review. This is mainly due to the fact that proposals submitted for expedited review are judged to be of minimal risk of harm, that study design is often less complex and that a smaller number of reviewers is involved. Assessing the timeline of the review procedure is a relevant indicator of ERB performance and dispels the myth that the ERB is responsible for a slow review process. Also, the overall time to approval should not be seen as the “time to getting a stamp”, but as a time of dialogue and critical reflection on both sides. In addition, ERB timelines are shortened in case of extraordinarily urgent situations and turnaround can occur within a few days.

To further improve its decentralized and very reactive way of functioning, a web-based platform was created exclusively for the MSF ERB. This platform provides access for ERB members to all routine communications, active files and ERB archives including all past ERB reviews, protocol amendments, research reports, ERB decisions, relevant MSF or ERB policies.

In the rest of this paper we outline five different innovations that have resulted in proportionate ethics review, without compromising on the fundamental role of the ERB.

### A new framework to guide ethics review

A new framework to help guide ethics review was adopted by MSF in November 2013. This replaced a previous ethics framework that had been adapted from the work of Emanuel et al. [6]. In this section we will explain both what is different about the new framework and why the changes were made. However, we will begin by explaining why a framework is necessary at all. What is an ethics framework? What is its role? How does it work?

An ethics framework can serve many purposes. First, it seeks to articulate and make explicit the kinds of values or other ethical considerations that are taken to be of crucial importance in relation to the topic in hand, in this case, the ethics of research conducted in the course of humanitarian medicine. Any framework will be the product of a particular time, influenced by the knowledge and experience of those involved in producing it. Articulating the considerations that are held to be relevant allows for transparent discussion, critique and development of the framework over time in the light of evolving situations. Second, and perhaps most crucially, an ethics framework provides assistance to researchers and others that evaluate and/or make decisions about the ethics of research. This means that a framework will often be an aid to research design and deliberation, not just a theoretical expression of ideals. On this view, it ought to be of use to members of the ethics board, but also of aid to researchers and anyone else thinking about the relevant issues (as the document is publicly available on the MSF website [7]). Third, it is also important to recognise what a framework does not do. It does not, for example, determine what should or should not be done. It is not a piece of legal or quasi-legal regulation. Rather, its intent is clearly expressed by the metaphor of ‘framing’; it aims to ‘frame’, or provide an outline or structure for a focused and relevant discussion and eventual decision, not to provide a list of necessary and absolute principles or statements.

The original MSF ERB framework was based on the work of Emanuel et al. This was used, because at the time it was the most practically orientated of the various frameworks, guidelines and documents available. It provided a coherent summary of the issues that were central to discussions of research ethics, building upon the many documents then available [8-10]. The Emanuel framework seemed to synthesize these diverse guidelines into something tangible. Despite its limitations briefly described below, it provided a focus for both reviewers and researchers. It is fair to say that the new ethics framework is a development from the work of Emanuel et al., but it is significantly different in a number of ways.

There were three main motivations for developing a new framework. First, and perhaps most importantly, the Emanuel framework actually provides little practical guidance. It is more of a checklist of important considerations than a framework that facilitates making ethical judgments. For example, the framework is structured in terms of a series of ethical ‘principles’ and related ‘benchmarks’. However, it is unclear what a benchmark is. How do the different ‘benchmarks’ relate to each other? What is the moral status of a benchmark? Is it an ethical principle, a value or just a statement? What practical priority is to be given to each principle or benchmark? How do the different principles and benchmarks relate to each other and what should we do if they conflict? Is the presented order of the principles and benchmarks important or not? For example, the old framework’s first stated ‘principle**’** is ‘Collaborative Partnership’, followed by ‘Social Value’ and then ‘Scientific Validity’. In what sense are these supposed to be principles? They might be things that researchers and the ERB are supposed to ‘take into account’, but how do they do this? Is “Collaborative Partnership” the most important ‘principle**’**? Is that why it is first? Emanuel et al. [8] suggest that their requirements are presented in chronological order, although the detail of the order could be questioned. For example, why is a procedural requirement (independent review) in the list, and why does it appear where it does? Why is informed consent prior to, and separate from, respect for potential and enrolled subjects? If we consider the ‘benchmarks’ the first thing that is noticeable is that they are very didactic (Table 1).

**Table 1:** **An example of the ‘Benchmarks’ Linked to a ‘Principle’ in the old framework**

**Principle**	**Benchmarks**
Collaborative Partnership	1) Engage in partnership with national and/or international research institutions as relevant and appropriate.
	2) Collaborate with local and national researchers and health policymakers to share responsibilities for determining the importance of health problem, assessing the value of the research, planning, conducting, and overseeing the research, and integrating the research into the health system.
	3) Respect the community’s values, culture, traditions, and social practices.
	4) Involve the community in which the study takes place (hereinafter referred to as “study community”) through a consultative process in designing the research, in its implementation (advice on problems occurring during study, feedback of intermediate results) and in assessing how research results may be made beneficial.
	5) Contribute to developing the capacity for researchers and health policymakers to become full and equal partners in the research enterprise.
	6) Share fairly the financial and other rewards of the research.

The benchmarks state what should happen in a firm and clear way. There is a mixture of useful advice about engaging with local communities and researchers, although it remains unclear *why* this should be done. Is it for practical reasons e.g., because this is the best way to get the study results? Or is it for ethical reasons? If so, it is unclear what they might be, especially as ‘collaborative partnership’ is not an ethical principle. For example, you might expect some possible commitment to ideas of respect, or equality and justice. Second, the benchmarks are not always helpful. For example, consider the benchmark: ‘Respect the community’s values, culture, traditions, and social practices’. What does this mean? Does this relate to the kinds of issues just mentioned, such as a commitment to equal respect and value of persons? Are we to take it literally as a general didactic statement? Is it a commitment to some form of relativism? Are we to commit ourselves to engage with and effectively endorse any existing injustices in the population of study? If women have a marginalised role in that society, should we ‘respect’ this? At the very least, these are complex matters where the benchmarks often fail to capture the nuances relevant to thinking about ethics. Similar kinds of questions can be asked of many other research ethics frameworks and guidelines, as it is often left obscure how they are to be used as practical tools [11,12].

Second, the academic literature and debates have proliferated and developed over the last ten years, especially in relation to post-research benefits for host populations. Many of these discussions are of particular importance for research conducted by MSF. MSF as a medical organisation is providing health care to vulnerable and destitute populations and is devoting limited resources to research with the sole aim of improving health care. Third, there has been a growing awareness that the kind of research conducted by MSF may not be well served by traditional research ethics for several reasons: the chosen method is only rarely one of a randomised clinical trial of novel medicines (the model that has dominated research ethics) and is more likely to use retrospective analysis of previously collected clinical data, surveys, needs assessment or case control methods; the context for research is distinctive, in that much research is conducted in contexts of disaster, conflict, poverty, social exclusion and lack of access to care, disrupted health systems or other difficult circumstances, where there is great vulnerability and pressing humanitarian needs.

We believe that the new ethics framework [13] is an improvement in a number of ways. First, the overall structure of the framework is clearer. The issues are now placed into three groups (Research Question and Methodology; Respecting and Protecting Research Participants and Communities; Implications and Implementation of the Research Findings) to reflect a logical temporal order of things to consider before, during and after the research has been conducted. Second, this structure and, in particular, the use of questions should aid deliberation by anyone using the framework. Formulating the content of the framework in terms of questions very deliberately ‘frames’ the discussion about research ethics. It provides an open and flexible approach, rather than seeking to articulate a number of general statements. Table 2 illustrates this by focusing on the issue of informed consent.

**Table 2:** **An example of the difference between the two frameworks: consent**

**Old framework**	**New framework**
1) Involve the study community in establishing appropriate recruitment procedures and incentives for the participants.	***(2.2) What are your plans for obtaining consent? *** A requirement to inform participants is often seen as being an important way to show respect and promote patient autonomy and welfare.
2) Ensure that consent procedures are acceptable within the study community (may include supplementary community and familial consent procedures).	
	a. What information ought to be provided? This will usually include the following elements: the reasons for doing research, details about who is doing the research, why the potential participant is being asked to be involved, details about what any intervention might involve and any on-going commitments of participation, details about anticipated risks and benefits, the fact that participants are free to refuse or withdraw, that any findings will be communicated back to the participants etc. The information given should be proportionate to any risks, but this does not mean that the higher the risk, the more information ought to be provided. Sometimes, calling attention clearly to a common or significant particular risk is more important than listing every possible remote risk.
3) Disclose information in culturally and linguistically appropriate formats. This implies that	
• any information given during the informed consent process must be pretested with people of a similar cultural and educational background as potential study participants;	
• the information provided on the consent form must be in simple language, avoiding technical terms;	b. Providing information does not guarantee it has been understood. How can information be provided at an appropriate linguistic level, without jargon or technical terms, and appropriate to the local language and culture?
• the consent form must be translated into the local language and then back-translated into the “international” language used to get a sense of the accuracy of the translation and correct mistakes;	c. Should information be provided in oral and/or written form?
4) Ensure that participants fully comprehend the research objectives and procedures:	d. How will the consent process be conducted? You may want to consider issues such as: who will consent, where they will do so (is the place appropriate to allow a confidential discussion), will a witness to the consent be required, how much time will be offered to consider whether to be involved? Prior engagement with communities can be a useful way to ensure that the consent process meets local expectations and sensitivities. How will the act of consent be recorded (e.g. signed and witnessed document, thumb print etc.)?
• if needed, the person should get time to discuss the information received with members of the community or family before deciding on consent;	e. Alternative or additional consent procedures may need to be developed where potential participants are minors, minor parents, or suffering from short or long-term incapacities etc.
• in addition, community information or “schooling” on the research to be done and on the purpose and process of seeking informed consent will raise pre-enrolment awareness and thus help people to decide if they want to participate in the study.	f. It should not be assumed that a long and complicated information sheet is always necessary and in exceptional cases it may be justifiable not to seek informed consent. Where researchers believe that this is appropriate, they should be careful to providereasons for this in the protocol.
5) Obtain consent in culturally and linguistically appropriate formats.	
6) Ensure that potential participants are free to refuse or withdraw from the research at any stage without penalty..	

The old framework sets out a set of commands as if they were absolute rules that must be followed. The new framework covers much of the same ground, but approaches the issues in a different spirit. It seeks to invite researchers to justify their approach. It accepts that sometimes it is inappropriate or impossible to gain a fully informed consent from every individual. Help is offered to the researcher, but the aim is to encourage a conversation about ethical issues, rather than impose quasi-legalistic rules. The questions help potential researchers to see what the ERB wishes to see in a protocol and ethics application, but can also help researchers come to understand ethical issues in more detail, and how ethical issues are intertwined with methodological issues. The use of open questions requires researchers to engage with the framework and outline and defend how they will address the relevant issues that they have identified. Ethics review is more of an active process, with the researcher as a participant, rather than the victim of an alien and abstract set of rules. Third, the new framework is more engaged with the specific MSF research context. This means that certain values/principles/issues are given much greater prominence than they ever have before. For example, it is now much clearer how methodological and ethical issues interact and the obligations that arise once research has been completed are much more prominent and require greater attention^b^.

The new framework has been implemented since November 2013 and has been well received by MSF researchers. It will be evaluated after 12 to 18 months’ use from the researchers and the ERB’s perspective.

### Exempting research based on a posteriori analysis of routinely collected data from review

MSF collects a vast amount of data as part of its routine clinical procedures. At a later stage these data may be useful to evaluate programme outcomes, analyse predictors of treatment outcomes, or assess factors influencing programme or treatment effectiveness. As the number of protocols aimed at generating this kind of data submitted to the ERB for review was steadily increasing, the possibility of delegating ethical responsibility for this type of study to the MSF medical directors under certain conditions was discussed at a joint meeting of the ERB and medical directors in June 2010. Primary considerations concerned (1) how these data were collected and (2) how patients were informed of the potential uses of their health data. Collecting routine clinical data should presumably follow universal ethical principles and thus might be thought to require consent. The ERB thus encouraged MSF to establish a policy on routine collection of data in clinical settings. The uses of such data may carry a low risk of harm, informing each patient may be difficult to implement and it is arguable that consent to at least some uses is implicit in clinical care. It was thus suggested that MSF consider a variety of ways of informing people about the possible uses of their data (e.g. well displayed posters describing the potential uses of health data collected in the clinic).

It was agreed that *a posteriori* analyses of routinely collected clinical data do not require ERB review, if MSF as an organization through the medical directors (who are overseeing research) takes responsibility for addressing the ethical issues. The following seven criteria must be fulfilled to qualify for exemption from ERB review:

1. Studies/articles are based on routinely-collected programme and clinical data.
2. They are either descriptive or targeted evaluations.
3. Confidentiality is respected; no individual patient identifiers are revealed.
4. Harm is minimal but acknowledged where relevant.
5. Potential benefits to both the programme and the community are described. Since the goal is publication, the relevance to a wider audience is described.
6. Collaborative involvement and, if applicable, authorship from a local medical or public health authority or partner (Ministry of Health, DHO, other NGO) is encouraged. If relevant and possible, consultation with a body representing the community is desirable.
7. If the decision for exemption from review is taken by the medical directors, the MSF responsibility to ensure that ethical requirements are met is similar to reviewed projects. This exemption, in addition, does not exempt MSF from compliance with regulatory requirements in the country from where the data originate. National or institutional ethical review may still be required.

Consequently between June 2010 and December 2013, 127 protocols involving exclusively a posterior analysis of routinely collected data were directly exempted from review by the medical directors. In case of doubt protocols are submitted to the ERB to ensure all criteria are met. This was the case six times in 2013. In all instances the ERB confirmed that exemption criteria were met. The list of exempted studies is provided to the ERB at the end of each calendar year. The ERB retains the right to audit exempted studies to verify that exemption criteria are respected.

### Preapproval of ‘emergency’ protocols

There is considerable uncertainty about optimal intervention in many dimensions of humanitarian medicine. Humanitarian interventions, as already noted, occur in an unpredictable manner and in uncertain contexts. Often the response must be implemented quickly and not according to the time table of meetings for established research ethics boards.

The need for rapid, yet robust, research ethics review has been noted in the literature. Experiences with SARS and pandemic influenza have demonstrated that existing research ethics structures are poorly tailored for rapid assessment and feedback in a sufficiently timely manner and may hinder legitimate research with potential benefits [14]. Recommendations for addressing these deficiencies have been tabled [15].

MSF frequently carries out research in emergency situations which do not allow sufficient time for prior ethics review. In a paper on MSF emergency research “external ethical review” is briefly mentioned with no indication if and how it is implemented [16]. The ERB has repeatedly been asked to conduct *a posteriori* reviews of emergency research in form of draft papers prior to publication. As emergency research may have serious implications for research participants, the issue of ethics review was revisited.

One recommendation that was adopted and passed by the MSF ERB was for pre-approval of generic protocols. Infectious disease outbreaks are a good example of the type of situation where such pre-approval can take place. There are often well structured research questions that are supported by the evidence as being a priority for answering through rigorous research. Protocols have been developed, but there is uncertainty about where the next outbreak will occur.

MSF ERB approved the following process:

1. When researchers have decided what topic to research in the next emergency, a “generic” research protocol is submitted to the ERB for review and pre-approval before the exact location is known.
2. Once the location is known, the final research protocol must be submitted to local ethics committee/authorities for approval.
3. At the same time, the final proposal is submitted to the MSF ERB, including details pertinent to the chosen location.
4. This review can be expedited by decision of the chair. In this case, the chair plus two members will pledge to provide a review and decision within 48 hours.
5. This decision can, however, be challenged by one or more ERB members, leading to full ERB review. It is expected that if this occurs, all members will provide input within an additional 24 hours.

This process has been successfully used in assessing the validity of new rapid diagnostic tests during a meningitis outbreak with no reported harms to participants and enhanced ability of researchers to respond in a timely manner. Until recently this was the only instance in which this process was used since it was agreed upon in 2008. The procedure has, however, been applied to a qualitative research protocol to understand local considerations and practices related to Ebola Virus Disease (EVD).

Emergency ethics review was also discussed for research to be carried out in the next Ebola fever outbreak. Previously, a review of research on filovirus haemorrhagic fever outbreaks (Ebola/Marburg) published between 1999 and 2007, had shown that among 34 research interventions, individual consent was sought in fifteen cases and ethics review (international and local) was mentioned only in three cases [17]. As MSF wished to carry out research on supportive treatments in major outbreak settings, the ERB suggested in 2010 a “generic” protocol be prepared, taking into consideration how to involve potential victims (or survivors) of these outbreaks in the development of such protocols. As outbreaks are unpredictable in terms of timing and location, finding individuals at the time might be unrealistic. However, some survivors are health workers and it may be possible to involve them in protocol development. In this specific case the “generic” research protocol could also be pre-approved by the relevant ethics committees of the countries concerned such as DRC, Republic of Congo, Gabon, South Sudan, and Uganda. This had not been taken forward by MSF before the recent Ebola outbreak in West Africa. ERB members, MSF medical directors and researchers met in October 2014, and subsequent decisions to conduct clinical trials in the Ebola outbreak were informed by the interaction of the ERB and MSF. The virtual structure of the ERB provided rapid and thorough expert review. It may also be noteworthy that a number of the MSF ERB members have also advised WHO on this issue.

### General review of “routine surveys”

One major question has repeatedly been where to draw the line between research and activities such as prospective disease prevalence surveys and rapid health assessments. MSF routinely carries out vaccination coverage surveys, nutritional surveys and retrospective mortality surveys. These surveys and assessments often have to be done in emergency settings for the purposes of program planning and emergency response, and historically have rarely been submitted to the ERB. Although these could be considered low-risk surveys, the same survey may be more sensitive in certain populations, such as a mortality survey in Iraq or in Syrian refugees, mapping of water quality in slums during the cholera epidemic in Haiti or assessing access to health services for undocumented migrants. Surveys cannot be presumed upfront to be of minimal risk. Many sensitive and morally problematic issues can be disclosed by surveys. Examples include discrimination or blaming of population groups, psychological distress, dealing with uncovered child abuse, or providing demographic characteristics of a community that may enhance their attractiveness as targets by combatants.

It was decided recently that the ERB should provide guidance without reviewing every survey, adopting a similar approach to that related to the retrospective analysis of routinely collected data. The ERB would trust researchers to deliberate about potential risks and whether they may want advice or review, within the following frame of reference:

- Standard vaccination coverage, nutrition and mortality survey instruments will be examined by the ERB and review exemption granted under certain conditions;
- Criteria for review exemption will be similar to those for *a posteriori* analysis of routinely collected data;
- Adding (or removing) a question from the standard protocol means that it is not a standard protocol anymore. Consequently an ERB review will be needed.
- Sensitive survey research, such as sexual activity, illegal behaviour or mental health, can never be exempted from ethics review.
- As for *a posteriori* analysis of routinely collected data, national or institutional ethical review in the country from where the data originate may still be required.

Implementation of this new policy will start in 2014, including submission of generic survey protocols to the ERB.

### Looking at the impact of approved research

While Principle 34 of the Declaration of Helsinki [8] only requests researchers to make provisions for post-trial access for all participants who still need an intervention identified as beneficial in the trial, the general wording in principle 20 (“… this group should stand to benefit from the knowledge, practices or interventions that result from the research”), might be interpreted in the sense of post-research access to a broader population. The CIOMS guidelines (Guideline 10) emphasise that “investigators must make every effort to ensure that the research is responsive to the health needs and the priorities of the population or community in which it is to be carried out; and that any intervention or product developed, or knowledge generated, will be made reasonably available for the benefit of that population or community” [9]. More recently, global actors have highlighted the need to ensure that the outcomes and benefits of publically-funded research are understood and mapped, and that a strategic approach to harnessing research results is taken by those seeking to improve healthcare delivery [18,19]. MSF as a global humanitarian actor doing research clearly has to ensure that the benefits of the research are made available to the study community. However, the responsibility of MSF goes beyond responding to the health needs of the research population, using positive results of research to improve policy and practice at the national and international level [20].

The ERB has always closely examined the social value of research, meaning its potential benefits for research participants, the community and beyond during the review process. To assess if claims made in research protocols have translated into practice, the ERB requested MSF for the first time in 2010 to report on the dissemination of research results and programmatic impact of all research approved since the inception of the ERB. We distinguish between impact in the literature (scholarly impact) and impact in the field of intervention. Although examining impact in the literature may have some value, one should not forget that what happens after publication is what is important with operational research and for organisations such as MSF. Changes in policy and practice at the national and international level and real effects on programme performance (changes to programme outcomes, decrease in morbidity and mortality) are more relevant than scholarly impact.

Dissemination of research results was categorized as (1) internal MSF report only, (2) limited sharing of results at national or international level and (3) peer-reviewed publication. The ERB received feedback on 234 protocols out of 248 reviewed between 2002 and 2013. Overall 11 studies had been refused by the ERB and 38 protocols had to been cancelled after approval. 80 research protocols were still ongoing at the end of 2013. Of the remaining 105 studies, 76 lead to one or several publications, 9 to a more limited dissemination of results (i.e. poster or oral presentations), while the results of 20 studies were only shared internally through a report.

**Figure 4:**
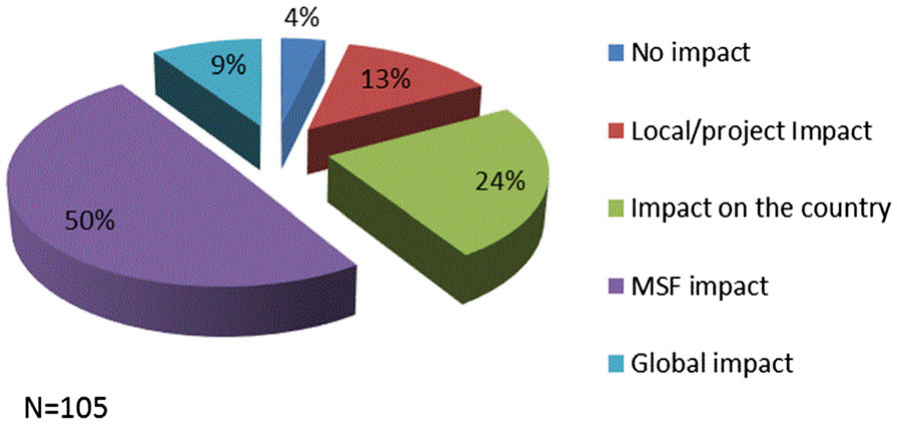
**Estimated impact of all studies submitted to ERB review and completed between 2002 and 2013.**

To assess impact at the programmatic and/or policy level four broad categories were proposed: local project impact, impact in the country where the study was done, impact within MSF, (potential) changes on a global scale. Given the fact that MSF research spans from opportunistic analyses of collected data to clinical trials, these categories do not necessarily imply a hierarchy. Some studies may be designed for local impact only; others may from the outset have the ambition to change therapeutic protocols internationally [21]. In Figure 4 below, each subsequent category encompasses the previous one. For example, impact at national level includes impact at the local level, and impact at a global scale comprises impact on MSF operations. Table 3 provides some examples for each of the four impact categories [22-30].

**Table 3:** **Examples of impact at various levels**

**Protocol title (date)**	**Outcomes**	**Publication**
**Global impact**		
Nifurtimox-eflornithine combination, human African trypanosomiasis, République du Congo (Clinical equivalence study comparing the nifurtimox-eflornithine combination with the standard eflornithine regimen for the treatment of Trypanosoma brucei gambiense) (2003)	Combination treatment now standard in MSF; stimulated DNDi to do the NECT study published in 2009. (Priotto G et al. Nifurtimox-efl ornithine combination therapy for second-stage African Trypanosoma brucei gambiense trypanosomiasis: a multicentre, randomized, phase III, non-inferiority trial. Lancet 2009; 374:56-64.) This led to change in WHO guidelines.	Priotto G et al. Nifurtimox-Eflornithine Combination Therapy for Second-Stage Trypanosoma brucei gambiense Sleeping Sickness: A Randomized Clinical Trial in Congo. Clinical Infectious Diseases 2007; 45:1435–42.
Mental health treatment outcomes in a humanitarian emergency. The evaluation of a pilot model for the integration of mental health into primary care in Habilla, Darfur (2009)	Tremendous success in sharing mental health tools among humanitarian actors	Souza R, Yasuda S, Cristofani S. Mental health treatment outcomes in a humanitarian emergency: a pilot model for the integration of mental health into primary care in Habilla, Darfur. Int J Ment Health Syst. 2009;3(1):17.
Tele-medicine/Tele-consultation - Does this service improve health care delivery in a remote conflict setting in Somalia? (2011)	The introduction of telemedicine significantly improved quality of paediatric care in a remote conflict setting and showed that this technology can be vital for training and capacity building. It has led to the expansion and use of telemedicine in similar settings. The study has also featured as a case example in the WHO World Health Report 2013.	Zachariah R et al. Practicing medicine without borders: tele-consultations and tele-mentoring for improving paediatric care in a conflict setting in Somalia? Tropical Medicine and International Health 2012; 17(9) 1156–1162.
**Impact within MSF programmes**		
Outcomes of a diabetic care program in Cambodia: an observational cohort study (2008)	Showed good blood sugar control could be achieved in a low-resource setting. Model of attaching chronic disease to HIV program unique. May be a useful model for other MSF programs looking at chronic diseases.	Raguenaud ME et al. Treating 4,000 diabetic patients in Cambodia, a high-prevalence but resource-limited setting: a 5-year study. BMC Med. 2009;7:33. doi:10.1186/1741-7015-7-33.
Compliance and diagnostic profile of referrals from Community Malaria Volunteers to the MSF supported health structures in Bo and Pujehun districts, Sierra Leone (2009)	Highlighted low levels of community based referral completions and its implications at community level. Feeds into future operational strategies related to use of community workers in malaria care	Thomson A et al. Low referral completion of rapid diagnostic test-negative patients in community-based treatment of malaria in Sierra Leone. Malaria Journal 2011;10:94.
Surgical site Infection after caesarean section: A proxy for problems in surgical care		
	Provided information on ways forward to improve post-operative case and improve vigilance on post-operative infection	Chu K et al. Caesarean section rates and indications in sub-Saharan Africa: a multi-country study from Médecins sans Frontières. PLoS One. 2012;7(9):e44484.
**Impact at country level**		
Drug efficacy trial of three artemisinin-based combination therapies: Artesunate + Sulfadoxine-Pyrimethamine, Artesunate + Amodiaquine and Artemether + Lumefantrine (Coartem) for the treatment of uncomplicated Plasmodium falciparum malaria, Republic of Congo (2004)	Led to artemisinin combination therapies becoming the national malaria treatment policy	van den Broek I., Kitz C., Al Attas S., Libama F., Balasegaram M., Guthmann J-P. Efficacy of 3 artemisinin combination therapies for the treatment of uncomplicated *Plasmodium falciparum* malaria in the Republic of Congo. *Malar J 2006* Nov 24;5:113.
Assessing home based treatment and care of MDR-TB patients in northern Uganda (2011)	Findings used for advocacy report to push for implementation of ambulatory treatment in Uganda. Ambulatory model now part of national protocol.	Poster: Casas EC et al. A decentralized community-based MDR-TB model of care in northern Uganda. MSF-UK Scientific Day, 25 May 2012, London, U.K and 43rd Union World Conference on Lung Health.
**Local level impact**		
Reasons why women default from a prevention of mother to child transmission of HIV (PMTCT) program and views of men on PMTCT activities in the informal settlement of Kibera, Nairobi, Kenya (2007)	Helped to generate knowledge on factors associated with default and re-orient the existing programs to improve community acceptability	Kizito KW et al. Lost to follow up from tuberculosis treatment in an urban informal settlement (Kibera), Nairobi, Kenya: what are the rates and determinants? Trans R Soc Trop Med Hyg 2011; 105: 52-57.
A case study of a collaborative initiative between an HIV/AIDS Clinic and a Community Non-Governmental Organization Network in Mumbai, India (2010)	The Mumbai team learned an important lesson regarding followup of defaulters: not to approach the homes, but to contact by other means. To avoid stigma.	Errol L et al. Tracing patients on antiretroviral treatment lost-to-follow-up in an urban slum in India. J Adv Nurs 2012; 68(11); 2399-409.

The new framework explicitly asks for the dissemination strategy at community and global level, including dissemination if the research findings are negative. In addition, post-research obligations must be articulated at the level of research participants, the community, others in the same situation elsewhere, and should include an (advocacy) plan in place to assure access to benefits of the study results beyond MSF’s and MSF partners’ engagement. Impact reporting has been systematised on an annual basis for all research protocols approved by the ERB. It is important to note that this approach to impact does not just follow orthodox approaches such as, for example, citation counts. The ERB has also encouraged MSF to share negative findings arising from MSF research as well as positive findings. However, this has not been explicitly assessed.

## Summary

This paper shares the experience of an independently functioning ethics review board serving a large medical humanitarian organisation and shows how it has evolved over time to face the growing challenges of research carried out with vulnerable communities in resource- constrained or emergency settings. First and foremost, we have noted over the years the importance of the dialogue between MSF researchers and independent ERB members. Continued interaction has allowed for progressive innovations based on a trustful and respectful partnership between the ERB and the researchers. During those years we have learned to work together and to better serve each other’s needs thus leading to a co-evolution of researchers and the research ethics board. And most importantly we have come to understand that research ethics should be seen as an iterative evolving process. Our experience shows that ethics review can be a constructive learning process, and a time of reflection and critical debate. We think research ethics review should take this format. This, of course, differs from the ordinary perception of institutional ethics review as legalistic, formulaic and at times obstructive.

While pursuing developments in various areas as described in this paper in the coming years, we would particularly like to focus our attention on three areas.

First, the impact of any new framework should be continuously assessed. While, in this case, we can already sense after six months implementation, that it has been adopted very easily and seems to be much more “intuitive” than the previous framework, a more rigorous evaluation of its acceptance, implementation and impact on the quality of research protocols would be useful not only for MSF, but also for other organisations that are facing the same challenges and might wish to adopt and/or adapt it.

Second, the impact of research needs to be defined more precisely to be meaningfully assessed. One must also consider that impact changes over time - short, medium and long term cumulative impact. The latency of the impact may be shown in a meta-analysis or meta- synthesis at a later time, or demonstrated by uptake of the results into guidelines. It is impossible to predict upfront when key impact will occur. Another important question will be how sustainable changes implemented based on research outcomes are over time.

Finally, the dialogue between the MSF ERB and the ethics committees in the study countries should be enhanced [31]. Until now this relationship has been limited to seeing each other’s approvals and sometimes the full comments. A more structured and continuous exchange could, for example, be initiated with national ethics committees in countries where MSF has been present a long time and is repeatedly carrying out research activities.

We hope that the innovations in research ethics governance described in this paper may be relevant for other organisations carrying out research in fragile contexts and for ethics committees reviewing such research.

## Endnotes

^a^Full review requiring participation of all ERB members, is warranted if the effectiveness, efficacy or safety of a given procedure or therapy is tested on human subjects and/or if the research involves collecting body/tissue samples with hypothesis testing (e.g. all clinical trials and some operational research projects). Expedited review, requiring participation of two or three ERB members, is deemed sufficient if the research carries only minimal risks to human subjects.

^b^A research ethics framework for similar contexts was produced by R2HC [32] after we had revised our framework. It will take us too far from the purpose of this paper to offer any detailed comparative analysis but it should be noted that the RCHC framework largely summarizes existing literature and follows our revised framework in many respects.
